# Versatile annotation and publication quality visualization of protein complexes using POLYVIEW-3D

**DOI:** 10.1186/1471-2105-8-316

**Published:** 2007-08-29

**Authors:** Aleksey Porollo, Jaroslaw Meller

**Affiliations:** 1 Department of Environmental Health, University of Cincinnati, Cincinnati, OH 45267, USA; 2 Department of Biomedical Engineering, University of Cincinnati, Cincinnati, OH 45267, USA; 3 Division of Biomedical Informatics, Children's Hospital Research Foundation, Cincinnati, OH 45229, USA; 4 Department of Informatics, Nicholas Copernicus University, 87-100 Torun, Poland

## Abstract

**Background:**

Macromolecular visualization as well as automated structural and functional annotation tools play an increasingly important role in the post-genomic era, contributing significantly towards the understanding of molecular systems and processes. For example, three dimensional (3D) models help in exploring protein active sites and functional hot spots that can be targeted in drug design. Automated annotation and visualization pipelines can also reveal other functionally important attributes of macromolecules. These goals are dependent on the availability of advanced tools that integrate better the existing databases, annotation servers and other resources with state-of-the-art rendering programs.

**Results:**

We present a new tool for protein structure analysis, with the focus on annotation and visualization of protein complexes, which is an extension of our previously developed POLYVIEW web server. By integrating the web technology with state-of-the-art software for macromolecular visualization, such as the PyMol program, POLYVIEW-3D enables combining versatile structural and functional annotations with a simple web-based interface for creating publication quality structure rendering, as well as animated images for Powerpoint™, web sites and other electronic resources. The service is platform independent and no plug-ins are required. Several examples of how POLYVIEW-3D can be used for structural and functional analysis in the context of protein-protein interactions are presented to illustrate the available annotation options.

**Conclusion:**

POLYVIEW-3D server features the PyMol image rendering that provides detailed and high quality presentation of macromolecular structures, with an easy to use web-based interface. POLYVIEW-3D also provides a wide array of options for automated structural and functional analysis of proteins and their complexes. Thus, the POLYVIEW-3D server may become an important resource for researches and educators in the fields of protein science and structural bioinformatics. The new server is available at .

## Background

Computational tools for protein structure visualization, analysis and functional annotation are being constantly developed and improved in order to enable better integration with quickly evolving proteomic databases and other on-line resources, to facilitate and automate annotation tasks of ever increasing complexity, and to address the growing demand for high quality structure rendering. In regard to the latter, emerging new technologies gave rise to a number of advanced, stand-alone tools for macromolecular graphics, including the PyMol program [1] that combines the beauty of modern graphics libraries with the power of the Python programming language for complex rendering commands. At the same time, web-based protein structure visualization and annotation resources have been gaining in popularity, partly due to the availability of public domain Web browser plug-ins, such as Jmol [2] or Chime [3]. A wide collection of links to both public domain tools and commercial software for macromolecular structure analysis and visualization can be found at World Index of Molecular Visualization Resources [4].

While stand-alone packages, such as MolScript [5], RasMol [6], YASARA [7], VMD [8], Swiss-PDBViewer [9], or PyMol [1], provide a wide range of functions for structure analysis and visualization, their use may be somewhat tedious, especially for non-experienced users. In particular, they often require some scripting and programming skills to optimize their use and generate high quality pictures with complex rendering. On the other hand, web-based interactive tools for the analysis of macromolecular structures are easier to use and less platform dependent. Many such tools, including PDBsum [10], PDB2MGif [11], Molray [12], AISMIG [13], or PPG [14], have been developed in the last several years, greatly facilitating and simplifying visualization and analysis of macromolecular structures. However, the capabilities of current on-line visualization resources and available annotation options require constant improvements. One trend is to enable generation of not only static pictures, but also animated movies, e.g., for analysis of macromolecular motion.

In this regard, the PDB2MGif server [11] should be noted as an early attempt in creating animated images for electronic resources in a fully automated way. However, PDB2MGif relies on the RasMol program [6] to generate the 3D rendering, with rather limited resolution and quality. Another example is the MovieMaker [15], which can be used for visualization and analysis of protein dynamics, utilizing MolScript and several other structure analysis tools. More recently, another tool for the generation of animated movies with improved resolution, multiple rendering options, and storyboarding capabilities, called PMG, has been developed [16]. In order to further address limitations of the current on-line tools in terms of the versatility, resolution and quality of images they can generate, and to specifically improve and streamline analysis of protein complexes and protein interaction interfaces, we have developed a new tool, called POLYVIEW-3D.

This new server represents a significant extension of our previous efforts to provide the proteomics community with a flexible web-based platform for protein structural and functional annotations [17]. In particular, POLYVIEW-3D integrates the ease of use of web-based tools with high quality models and structure rendering generated using the PyMol program [1]. In addition, POLYVIEW-3D couples publication quality visualization with advanced structure and function analysis, including mapping functional hotspots, such as known and predicted interaction interfaces, analysis of putative binding pockets (including those within protein interaction interfaces), comparison and scoring of protein docking models. These tasks are achieved by both specifically designed tools and using several state-of-the-art annotation and prediction servers that are coupled with POLYVIEW-3D, as outlined in the Implementation Section.

## Implementation

### Image rendering and animations

The primary input data format for the server is the standard Protein Data Bank (PDB [18]) format for macromolecular structures, with some extensions discussed below. For structures deposited in PDB, one may use their four letter codes to retrieve them automatically. In order to generate three-dimensional macromolecular models, POLYVIEW-3D utilizes primarily the PyMol program [1], which provides high quality rendering of 3D structure. These graphical representations, which would otherwise require complex rendering commands, can be generated easily by using specifically developed and tailored to common annotation tasks web interface.

For example, rendering using cartoon, wireframe, CPK, solid and transparent surface models, as well as their combinations for different molecules (e.g., protein *vs*. ligand) or their fragments (e.g., subsets of residues) are available through expandable, context dependent menu boxes. Several highlighting and coloring styles are also provided through such context dependent menus, complemented by the possibility of specifying multiple styles (e.g., colored according conservation and shown with or without surface rendering) for each residue by using a user defined highlighting list, with a convenient style converter and list manipulation tool. In order to further simplify the use of the server, the initial orientation, centering, and zooming of the molecule can be pre-specified or set interactively by using the Jmol program embedded into the POLYVIEW-3D web-interface.

In addition to static images, high resolution animated images can be generated as well, for inclusion in digital presentations and other electronic documents. In particular, such animations can be copied directly into a Powerpoint™ slide. For that purpose, standard and custom made PDB files with multiple models, such as NMR-derived structures, protein docking models in CAPRI [19] format, or molecular trajectories with snapshots of the system represented by subsequent models, can be used. Together with animated GIF files, all individual snapshots may be retrieved as well, with all the rendering, coloring and highlighting schemes being directly transferable between static images and animation movies. The resulting static images are available in PNG and TIFF formats, with a user defined resolution (both in terms of size and DPI), which enables generating publication-quality images of different sizes.

Since in some cases PyMol may require a substantial CPU time to generate complex 3D representations (e.g., when many animation frames are to be generated in high resolution for large macromolecules), a faster alternative that utilizes the RasMol program [6] is also provided. In addition, a quick preview function is provided for some types of queries. POLYVIEW-3D also integrates three-dimensional structure rendering with enhanced, high resolution 1D structure models that were previously available in low resolution versions through the original POLYVIEW server. Using these simple, yet versatile, structure representations that were developed using graphical functions available in an open-source graphics library *libgd *[20], often proves to be very useful in the analysis, guiding the generation of more complex 3D images.

We would like to point out that POLYVIEW-3D represents a major update of the original POLYVIEW server. While POLYVIEW is cross-linked and somewhat complementary with POLYVIEW-3D, and some of the simpler functions of the latter were recently integrated into POLYVIEW as well, these two tools are different in at least two fundamental aspects. First of all, POLYVIEW does not allow one to generate high quality rendering of 3D macromolecular structure, including animation movies. Secondly, POLYVIEW does not offer advanced annotation and analysis options that deal with topographical and other 3D aspects of macromolecules. On the other hand, POLYVIEW-3D is specifically designed to provide these capabilities, e.g., in the context of analysis of 3D characteristics of interaction interfaces, as described in the next section.

### Functional and structural annotation

There are several different types of annotation options for structural and functional analysis that are available in POLYVIEW-3D. Some basic options that were mentioned before include: highlighting various amino acid properties and their distributions within protein structure; displaying crystallographic temperature factors (if available) to identify flexible regions; generating animated movies to visualize conformational ensembles that are represented by multiple models in structures solved using NMR, or to visualize molecular motion as represented by Molecular Dynamics trajectories, distortions along Normal Modes (e.g., calculated using the Elastic Network Model approximation, as implemented in some on-line servers, such as AD-ENM [21]), or other macromolecular conformational changes (e.g., those available from the Database of Macromolecular Movements [22]).

Some more complex tasks, geared up primarily towards the interrogation of protein complexes and interaction interfaces, are achieved by combining several tailored tools, stand-alone software packages and web servers. These tasks include identifying and mapping known interactions found in protein complexes deposited in PDB, the assessment and mapping of evolutionary conservation onto the protein structure model, mapping and analysis of pockets in the structure as putative targets for ligand docking, and analysis and ranking of protein docking models. In particular, the DSSP program [23] is used for the identification of secondary structure elements, solvent accessibilities and residues at protein-protein interaction interfaces within protein complexes. The latter is specified in terms of changes in the exposed surface area of a residue upon complex formation, with default parameters defined and evaluated in [24]. Structures deposited in PDB can be processed on the fly in order to generate putative biological units from asymmetric units, using the PQS server [25]. In addition, PFAM domains may be automatically mapped into the query structure as well, by using sequence-based search implemented in the PFAM server [26], and subsequently visualized using a custom coloring scheme.

Furthermore, by using the SPPIDER server [24], which is coupled with POLYVIEW-3D, one can identify all PDB complexes (including biological units) that contain close sequence homologs of the protein of interest. Interaction sites from these alternative complexes can be then mapped to the query protein sequence (and thus structure) by using sequence alignment, as described in detail in [24]. Moreover, by combining evolutionary and structural (derived from an unbound protein structure) information, SPPIDER provides rigorously evaluated and extensively tested (including on a set of CAPRI targets) predictions of putative interaction sites, that were shown to achieve accuracies competitive with state-of-the-art methods [24]. These predictions are used here for the assessment of protein docking models, e.g., generated in the context of CAPRI evaluation. While other prediction servers (e.g. ConSurf [27]) can be used as well, at present POLYVIEW-3D fully automates this process only in the case of SPPIDER.

Specifically, a custom PDB file with multiple models of a protein complex in the CAPRI format (e.g., generated by the ClusPro server [28]) may be submitted to POLYVIEW-3D, triggering SPPIDER predictions in the background for both chains that are docked. Unbound structures of these chains are used to predict putative interaction interfaces, which are then compared with interfaces observed in each model (see Section Results and Discussion). The fraction of residues within the interface in a given model that overlaps with SPPIDER predictions (averaged over both chains) provides a simple score to (re-) rank the models. In addition, the surface area, average hydrophobicity, and evolutionary conservation for each interface within these models are computed to provide a basis for further analysis and visualization.

**Figure 1:**
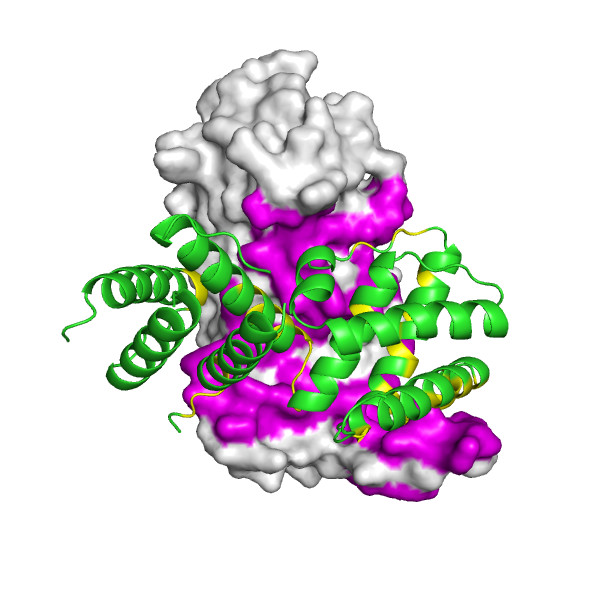
An example of POLYVIEW-3D visualization of an inactive form of the regulatory domain of LicT antiterminator (see text for details). The overall structure of the regulatory dimer is shown, with one chain shown using the surface, and the other chain using the cartoon rendering, respectively. The residues found to be within interaction interface are shown in magenta and yellow.

The evolutionary conservation may be assessed using the output from the ConSurf server [27], which yields well established relative (i.e., normalized within the query sequence) conservation scores. POLYVIEW-3D provides an option to upload ConSurf custom PDB files, with modified B-factor columns to represent these scores in terms of ConSurf coloring scheme. In addition, and in the context of known and mapped interaction interfaces, ConSurf provides the possibility to identify novel functional hot spots in terms of evolutionary conserved and surface exposed amino acid residues (see Figure 1C). As a simple alternative, POLYVIEW-3D also allows one to assess the evolutionary conservation in terms of entropy, which is derived from amino acid frequencies observed at a given position in a multiple sequence alignment (MSA). The latter is computed as a background process, using three iterations of the Psi-Blast program [29], with default options and the *nr *protein database [30]. Such generated estimates of variability (see the on-line documentation for details), and the underlying MSAs, contribute to the prediction of putative interaction interfaces used here for the analysis of protein docking models. While very useful in the analysis of MSA-based predictions, this approach, however, has not been fully tested as a model of divergence within protein families.

Another type of analysis, which is of interest in the context of drug design, is the identification of structural pockets and cavities in proteins, including those within interaction interfaces, as potential targets for docking studies. For that purpose, POLYVIEW-3D is coupled with the CASTp server [31], which is a well established on-line tool for the recognition of potential binding and active sites in terms of surface topography. Specifically, POLYVIEW-3D can be used to automatically retrieve and display CASTp results, using again a variety of styles (see Figures 1B and 1D). We would like to comment that to the best of our knowledge, at least two of the complex annotation features discussed above, namely the mapping of interaction interfaces from homologs, and automated analysis and re-ranking of protein docking models, seem to be unique in the context of on-line visualization servers with advanced rendering capabilities.

### User interface

POLYVIEW-3D does not require any plug-ins and is expected to be fully functional under any platform and with any recent Web browser. Moreover, an intuitive graphical interface alleviates the need to learn an often complex syntax of commands and options available in programs utilized by the server to generate images and annotations. At the same time, the web interface offers a number of options to customize the output and tailor the analysis of the system at hand. In addition, a script is provided for advanced users for download in order to improve the image by using locally installed rendering software. The POLYVIEW server also provides extensive cross-linking with other public annotation resources, such as the PDB [18] and NCBI portals [32], the UniProt annotation database [33], and various protein structure analysis and visualization tools. Detailed description of rendering options and different types of functional annotations that are available in POLYVIEW-3D, as well as examples of static and animated images, are included in the on-line tutorial at [34].

## Results and discussion

In this section, we illustrate how POLYVIEW-3D server can be used for protein structure analysis in the context of protein-protein interactions. In particular, we show several specific examples of structure rendering and annotation for a homodimeric complex of regulatory units of the transcriptional antiterminator protein LicT, which regulates the expression of *Bacillus subtilis *operons involved in beta-glucoside metabolism [35]. All options discussed here can be specified using the POLYVIEW web-interface. We would also like to point out that animated versions of the images shown here can be easily generated using the server.

The regulatory units of LicT consist of two five helical bundle domains called PRD1 and PRD2, which adopt dramatically different relative orientations in inactive and active forms of the protein (PDB: 1TLV, 1H99, respectively). In the activated state, each PRD forms a dimeric unit with its counterpart in the other chain, burying, at the dimer interface, phosphorylation sites that are critical for regulation (conserved histidine residues). In the inactive state, a wide swing movement of PRD2 results in partial opening of the dimer, making the phosphorylation sites accessible on the protein surface [35]. This inactive form of the dimer, with essentially only PRD1 and PRD1' domains involved in the formation of the interaction interface [35], was used in the CAPRI assessment as Target09 [36], and is shown here in Figure 1.

**Figure 2:**
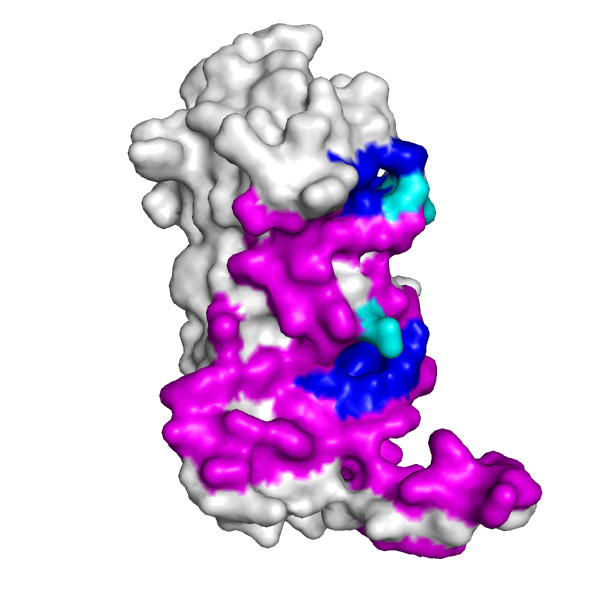
Chain A of the regulatory domain of LicT protein shown alone, with two largest pockets on the surface that partially overlap with the interaction interface (shown in magenta), as identified using CASTp, highlighted in blue and cyan (the latter for residues within the pockets that are also involved in the formation of the interaction interface).

**Figure 3:**
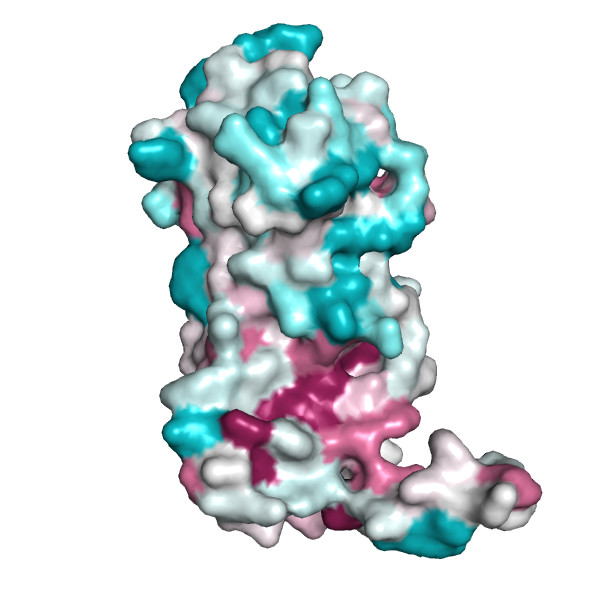
Chain A of the regulatory domain LicT protein shown alone, with surface exposed residues colored according to their evolutionary conservation, as assessed by the ConSurf server (residues that are highly conserved are shown in deep purple, whereas highly variable positions are shown in cyan).

**Figure 4:**
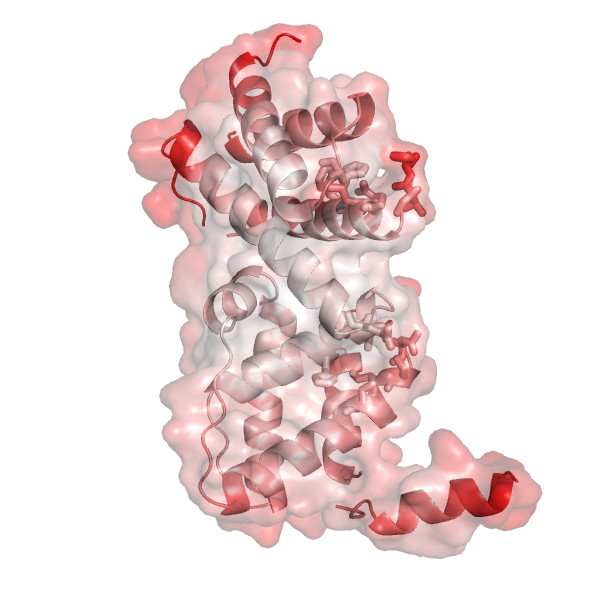
Same as Figures 2 and 3, with colors representing B-factors this time (red corresponding to highly flexible, and white to relatively rigid parts of the structure, respectively), and with the semitransparent rendering of the surface (residues forming pockets shown in Figure 2 are highlighted using stick models for their side chains).

As can be further seen from Figures 2, 3 and 4, protein chains can be rendered using different models, including available surface representations with different coloring schemes. In particular, we illustrate various structural annotations that can be automatically generated and displayed, including the identification of interacting residues within the complex (Figures 1 and 2), visualization of (two) large pockets partially overlapping with the observed interface (shown in blue and cyan in Figure 2) that were identified using CASTp, coupled with the analysis of evolutionary conservation and putative functional hotspots carried out using ConSurf (Figure 3), as well as estimated flexibility due to thermal motions, as encoded by temperature factors (Figure 4).

We would like to comment that a relatively large, rather hydrophobic with some hydrophilic hot spots (data not shown) and relatively rigid (in this bound structure) pocket that involves some conserved residues, is identified in the central groove of the structure, essentially in between two distinct patches forming the interface. Given the importance of the dimerization state and interface formation for the function of the protein, this pocket may represent a valuable target for ligand design. We would also like to point out that POLYVIEW-3D can be used for additional analysis of conformational changes between inactive and active forms by generating animated movies and individual snapshots of putative trajectories representing transition between the two structures, as generated, e.g., by the AD-ENM server [21], or obtained from the Database of Macromolecular Movements [22].

**Figure 5:**
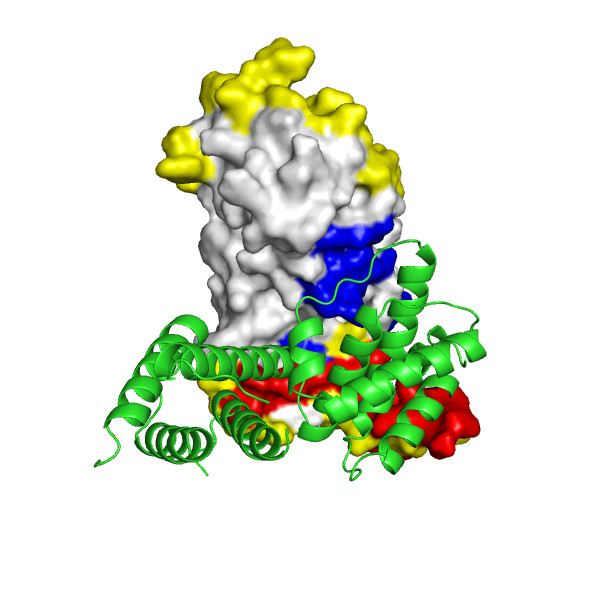
Application of POLYVIEW-3D to the analysis and further assessment (in terms of the overlap between predicted and observed interaction interfaces) of protein docking models, generated for LicT using the ClusPro server (see text for details). An overall view of the top scoring model is shown, with PRD1 domains forming a qualitatively correct dimer interface. The residues found to be within the interaction interface in this model, which are also predicted by SPPIDER as interaction sites, are highlighted in red, residues observed in the model within interacting sites and not predicted as such are shown in blue, and residues predicted to be interacting sites but not involved in interactions in the model of the complex are shown in yellow, respectively.

**Figure 6:**
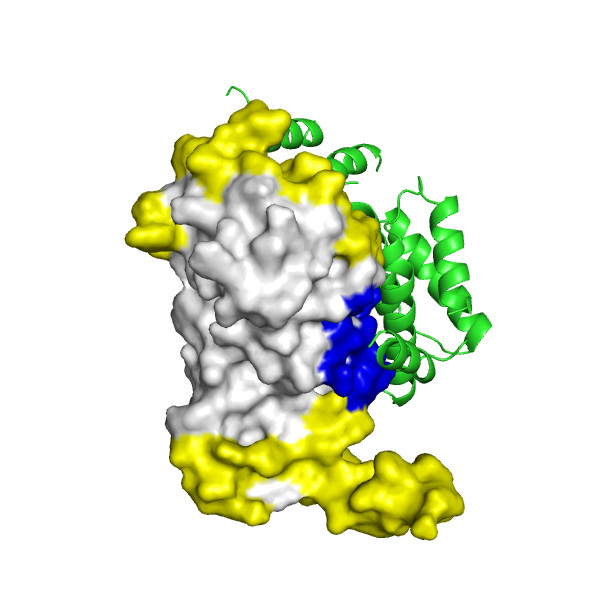
Visualization of an alternative (and qualitatively incorrect) ClusPro model for the LicT regulatory domain complex, using the same color scheme as in Figure 5. Note the lack of overlap with SPPIDER predictions in this case.

In Figures 5 and 6, we further illustrate the analysis and scoring of protein docking models, using an approach that was described in the previous section. Putative dimer models were generated for this system by the ClusPro server, and were submitted to POLYVIEW-3D. Two different models (ranked by ClusPro as number one and nine, respectively) are shown for comparison. The first model (Figure 5) is qualitatively consistent with the inactive form of LicT regulatory module, with PRD1 domains forming most of the interface, and PRD2 domains in an open orientation (although somewhat different than observed experimentally – see Figure 1). The other model chosen here for illustration purposes, and shown in Figure 6, is characterized by a very different (and incorrect) orientation of the monomers, and the resulting interaction interface.

The correct ranking of these two models by ClusPro is certainly very encouraging. However, in general, multiple and often vastly different models from protein docking simulations are difficult to assess. Therefore, contrasting the results of protein docking approaches with predicted functional hot spots and interaction interfaces, provides a complementary approach to further improve model ranking and confidence in the models selected as top candidates. Using POLYVIEW-3D greatly facilitates such comparative analysis. In this particular case, as can be seen from the figures, only the first model shows significant overlap with predicted (from unbound structures) interaction sites, which are highlighted in red (for residues observed within the interface in a given model, and predicted as interaction sites) and yellow (for the remaining predicted interaction sites).

We would like to comment that SPPIDER predicts in this case two distinct interaction interfaces in the N- and C-terminal regions of the regulatory domain, coinciding with PRD1 and PRD2 subdomains. While only one of these interfaces is present in the inactive form analyzed here in detail, due to the rearrangement of the structure in terms of the relative orientation of PRDs, the other predicted patch overlaps, in fact, with the alternative interface observed in the active form of LicT. The latter can readily be verified using POLYVIEW-3D and the mapping of interaction sites from multiple complexes involving close homologs of the chain of interest, as described in the previous section.

While this is just an example of an application of POLYVIEW-3D to simplify the analysis and visualization of protein docking models, and caution should be exerted to avoid over-interpretation of such results in any particular case, on average, similar level of accuracy was observed for other CAPRI targets [24]. In the future, we are planning to expand this option to include other types of potentially useful scoring functions, including user provided contact potentials and other measures for model assessment (see, e.g., [37]).

## Conclusion

We present a new Web-based server, called POLYVIEW-3D, for versatile structure annotation and high quality visualization (including static views and animations) of macromolecules, with the focus on the analysis of protein complexes and protein interaction interfaces. POLYVIEW-3D integrates and greatly simplifies the use of programs that are employed for structure analysis and image rendering. In particular, the new server features the PyMol rendering program, which is coupled with several rigorously validated annotation and prediction servers, such as ConSurf, CASTp, ClusPro, and SPPIDER, providing an easy to use platform for gaining insights into protein structure and function, and facilitating common analysis and annotation tasks. The new server offers a number of advanced rendering options for preparation of figures and electronic materials, complementing stand alone and existing web-based tools in that regard.

## Availability and requirements

• Project name: POLYVIEW-3D

• Project home page: 

• Operating system: Platform independent

• Programming languages: Perl, JavaScript

• Other requirements: Web-browser supporting W3C standards

• License: The use of the server and its results are freely available for all users; However, in the interest of better serving the community with limited computational resources, the authors reserve the right to limit the number of submissions per unit of time

• Restrictions to use by non-academics: Same as above

## Abbreviations

PDB, Protein Data Bank; NCBI, National Center for Biotechnology Information; DPI, dots per inch.

## Authors' contributions

JM and AP conceived of the study and designed the server functionality. AP implemented the server and JM performed structure analysis for use as an example in the paper. Both authors drafted the manuscript, read and approved the final version.
